# Challenges and coping experiences faced by nursing staff in long-term care facilities in China: a qualitative meta-analysis

**DOI:** 10.3389/fpubh.2023.1302481

**Published:** 2024-01-08

**Authors:** Shibo Zhang, Xixi Xiao, Yating Ai, Ailin Zhang, Chunyi Zhou, Hui Hu, Yuncui Wang

**Affiliations:** ^1^School of Nursing, Hubei University of Chinese Medicine, Wuhan, China; ^2^Engineering Research Center of TCM Protection Technology and New Product Development for the Older Adult, Wuhan, China; ^3^Hubei Shizhen Laboratory, Hubei University of Traditional Chinese Medicine, Wuhan, Hubei, China

**Keywords:** thematic analysis, qualitative research, long-term care, nursing staff, challenge, experiences

## Abstract

**Objective:**

The aim of this study is to discern the challenges and coping experiences encountered by nursing staff in long-term care facilities in China. This will be achieved through the identification, evaluation, and qualitative synthesis of comprehensive data.

**Design:**

This is a qualitative meta-analysis.

**Methods:**

The research systematically examined relevant literature sourced from six databases, concluding the search in August 2023. The inclusion criteria encompassed qualitative and mixed-methods studies in both Chinese and English, focusing on challenges faced by nursing staff in long-term care facilities and their corresponding coping strategies. The application of the Preferred Reporting Items for Systematic Reviews and Meta-Analyses (PRISMA) framework facilitated the qualitative meta-integration process. Three independent researchers meticulously screened and assessed the quality of the chosen studies. The synthesis process sought to amalgamate and structure analogous findings into novel categories through multiple readings of the original literature. These categories were subsequently distilled into comprehensive themes.

**Results:**

Analyzed 15 articles revealed 14 sub-themes and 4 overarching analytical themes. These encompassed Sources of Challenges such as multitasking, clinical emergencies, workplace conflict, demand exceeding resources, and occupational discrimination. Psychological impacts included suppressed emotion, compassion fatigue, and self-doubt. Practical consequences involved damaged health, imbalanced life, and occupational disappointment. Coping strategies identified were self-adjusting, feeling validation and belonging, and finding support.

**Conclusion:**

Our research identified the challenges faced by nursing staff in Chinese long-term care facilities and their coping experiences. We found that most challenges could be mitigated through appropriate adjustments in managerial strategies, such as reasonable human resources planning, and providing resource support, including material, emotional, and informational support. Similarly, institutions should have offered necessary emotional and psychological support to nursing staff to overcome the negative impacts of challenges and encourage them to adopt positive coping strategies.

## 1 Introduction

China grapples with a pronounced global challenge of an aging population. As per the findings from the seventh national population census in 2020, the population aged 65 and above in China had surged to 190 million, marking a 4.63% increase compared to 2010 (1). The ongoing expansion of the older adult demographic underscores manifold challenges in social, economic, and human resource management. In this context, the importance of nursing staff in long-term care facilities as the primary providers of care for the older adult was continually recognized. Nursing staff included registered nurses, licensed practical nurses, and nursing assistants, among others (2). In China, the main responsibilities of geriatric nurses included daily care, health monitoring, assistance with daily activities, and emotional support (3). The primary responsibility of nursing assistants was to assist geriatric nurses in providing daily life-related services for the older adult (4). Therefore, nurses were an integral part of the nursing staff. During the process of caring for the older adult, nursing staff might have encountered various challenges from the older adult, such as cognitive decline (5), psychological disorders (6), muscle weakness (7), difficulty swallowing (8), and more.

In general, according to existing research, challenges faced by Chinese Nursing staff mainly stemmed from a lack of professional knowledge and skills (9, 10), workload and time pressure (11–13), compensation and benefits (9, 14), institutional management (15, 16), and interpersonal relationships (17), among other factors. Particularly in the realm of interpersonal relationships, nursing staffs in Chinese older adult care seemed to face greater complexity. On one hand, the concept of “filial piety” deeply influenced people's behavior and attitudes in traditional Chinese culture (18). This concept emphasized the respect and obedience of juniors to seniors, undoubtedly affecting the mutual relationship between the older adult and nursing staffs. On the other hand, the prevalent notion in Chinese family culture of “men working outside, women managing the household” was widespread. Longer working hours might have squeezed the responsibilities that female nursing staffs had to shoulder in the family (19). Therefore, Chinese older adult nursing staffs not only needed professional skills but also required advanced communication abilities, caregiving skills, and the ability to balance work-family responsibilities (20). These challenges might have led to a range of negative impacts on nursing staffs, including decreased work efficiency (21), professional burnout (22), or increased turnover rates (2). Over time, this might have had serious consequences for the Nursing staff health (23), such as musculoskeletal disorders (24), anxiety, or depression (25).

The coping strategy involves individual behavioral methods of managing stress by adjusting physiological and emotional responses, reducing discomfort (26, 27). The challenges and coping strategies faced by older adult nursing staff in China have been extensively discussed. Research indicates that Chinese older adult nursing staff are more likely to adopt proactive coping strategies to deal with the difficulties they encounter in their work. This includes adjusting salary structures (14), gaining educational opportunities (28), receiving support from nursing managers (15), and ensuring job stability (29), among other factors. However, current research tends to focus more on the challenges and coping strategies of nursing staff in their work, with less attention given to the challenges, impacts, and coping strategies related to their professional needs, family responsibilities, and societal discrimination.

Until now, some qualitative studies have reported the work experiences of nursing staff in long-term care facilities. However, to our knowledge, qualitative research evidence has not been fully synthesized and analyzed. Quantitative systematic reviews emphasize the quantification of effects and relationships [such as correlations (30), acceptability (31), or the aggregation of influencing factors (32)], while qualitative systematic reviews prioritize the exploration and understanding of complex phenomena [such as experiences (33), perspectives (34), or coping strategies (35)]. Therefore, this study aims to analyze, from the perspective of nursing staff, the challenges and coping strategies faced in their work through the synthesis and integration of existing evidence. The main questions addressed include, “What challenges do nursing staff in Chinese long-term care facilities face? What impact do these challenges have on nursing staff? What consequences may arise? And what coping strategies do nursing staff adopt?” The ultimate goal for future researchers is to design targeted interventions to alleviate the psychological and physiological challenges faced by nursing staff.

## 2 Methods

### 2.1 Study design

The present study employed a thematic synthesis approach (36) and adhered to the guidelines set forth by the Preferred Reporting Items for Systematic Reviews and Meta-Analysis (PRISMA) (37) for reporting, which can be found in Supplementary File 1. Furthermore, the study protocol was duly registered in PROSPERO under the identifier CRD42023460195.

### 2.2 Search strategy

The electronic databases PubMed, Web of Science, Cochrane Library, CNKI, WanFang, and Vip were systematically searched from the inception of each database to August 2023. The search terms were divided into the following eight categories: nursing staff, long-term care, pressure, work experience, challenge, Countermeasures, qualitative research and China. The full search details are shown in Supplementary File 2. Additionally, the reference lists of the included studies were manually searched to retrieve all relevant studies.

### 2.3 Inclusion and exclusion criteria

#### 2.3.1 Inclusion criteria

Participants: Nurse or nurse assistant. Interest of phenomena: The real experience of nursing staff in experiencing and adjusting to challenges. Context: Institutions that provide long-term care for the older adult, such as hospitals, nursing homes, and community health service centers. Studies design: Qualitative studies and qualitative components of mixed-method studies (Qualitative data can be extracted from it). Language: Studies written in Chinese or English. Focus of studies: The study was conducted in China. Publication type: Full papers published in a peer-reviewed journal.

#### 2.3.2 Exclusion criteria

1. Systematic reviews and other reviews.
2. The full text cannot be obtained, and the data is incomplete.
3. Repeated or suspected repeated publication (including titles, results, publication journals, and similar information that is not entirely consistent), similar literature only includes the most comprehensive information.
4. Delete low-quality literature (JBI ≤ 5), The main feature is a lack of method description or unclear data.

### 2.4 Study selection

**Figure 1:**
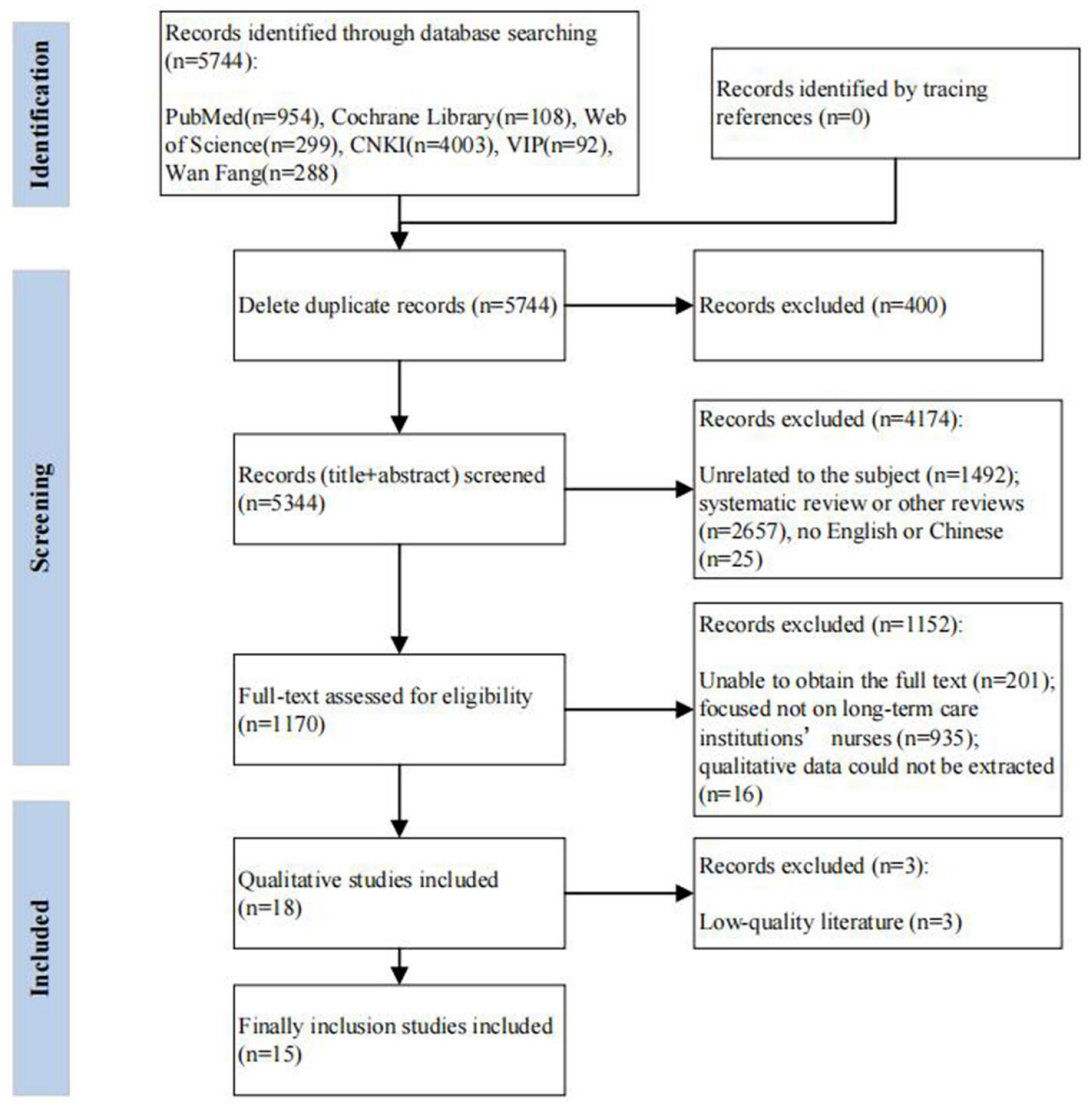
Flow diagram of studies included and excluded at each stage of review.

The search results have been imported into the reference management software EndNote X9. Initially, the researchers deleted duplicate records. Then, two researchers independently screened the titles and summaries of the articles and finally read the full texts for further assessment. Two researchers simultaneously screened the literature according to the inclusion and exclusion criteria. In case of differences, a third researcher was invited to discuss and resolve them. This flowchart is illustrated in Figure 1.

### 2.5 Quality appraisal

Two researchers utilized the Joanna Briggs Institute (JBI) Qualitative Appraisal Instrument to independently conduct a quality assessment of the article (38). This checklist comprises 10 items, with a total score of 20 points. Each item is assessed using “yes,” “no,” or “unclear” (Yes = 2, No = 0, Unclear = 1). If necessary, the third researcher was consulted for discussion of potential solutions. Refer to Supplementary File 3 for details.

### 2.6 Data extraction

The collected data was imported into NVivo 11 software for data extraction. BS conducted the data extraction, and cross-verification was performed through XX. Any discrepancies were resolved through discussions with YT. The extracted data includes authorship, publication year, country or region, research methods, research topics, research outcomes, and any views on challenges or coping experiences mentioned in the articles. These findings are primarily derived from the abstract, results, and discussion sections. Comprehensive data includes direct quotes from nursing staff, as well as the main researchers' interpretations of research results and key findings. It is noteworthy that if the data within the articles does not align with the theme of this study, it will not be extracted.

### 2.7 Qualitative data synthesis

The methodology is guided by the three-step thematic synthesis framework outlined by Thomas and Harden (36). Initially, SB and XX independently engage in line-by-line thematic coding, assigning “free codes” to elucidate the meaning and content of each line. Subsequently, YT conducts a secondary check of the “free codes” and their corresponding text to ensure their accurate representation. In the second step, BS and XX categorize and group the codes based on the similarity and dissimilarity of “free codes,” thereby forming descriptive themes. These descriptive themes serve to summarize and synthesize all “free codes” from the initial stage, with labels derived from the data and codes they encapsulate, endeavoring to maintain proximity to the original data. Explicitly, descriptive themes are categorized as either “challenges” or “coping experiences.” Lastly, to generate analytical themes extending beyond the data, BS and XX individually formulate these themes through continuous review, synthesis, and analysis of the descriptive themes. A subsequent group discussion among all authors is conducted to determine the final analytical themes, encompassing four aspects: sources of challenges, psychological impacts, practical consequences, and coping strategies.

The Confidence in the Evidence from Reviews of Qualitative Research approach (CERQual) is used to assess the confidence in the findings of qualitative research. CERQual consists of four components: methodological limitations, consistency, adequacy of data, and relevance. Methodological limitations refer to the evaluation using the JBI qualitative assessment tool. Consistency refers to the extent to which the results of individual reviews are supported by the primary studies contributing to them. Adequacy of data refers to the richness and quantity of data supporting individual review findings. Relevance is about how well data from primary studies align with the context specified in the review question. Two reviewers analyzed individual review findings based on these four components and discussed the confidence assessment with a third reviewer. This information can be found in Supplementary File 4.

### 2.8 Ethical considerations

This meta-analysis was carried out in compliance with the PRISMA guidelines and referred only to published data. Therefore, no ethical approval was required.

## 3 Results

**Figure 2:**
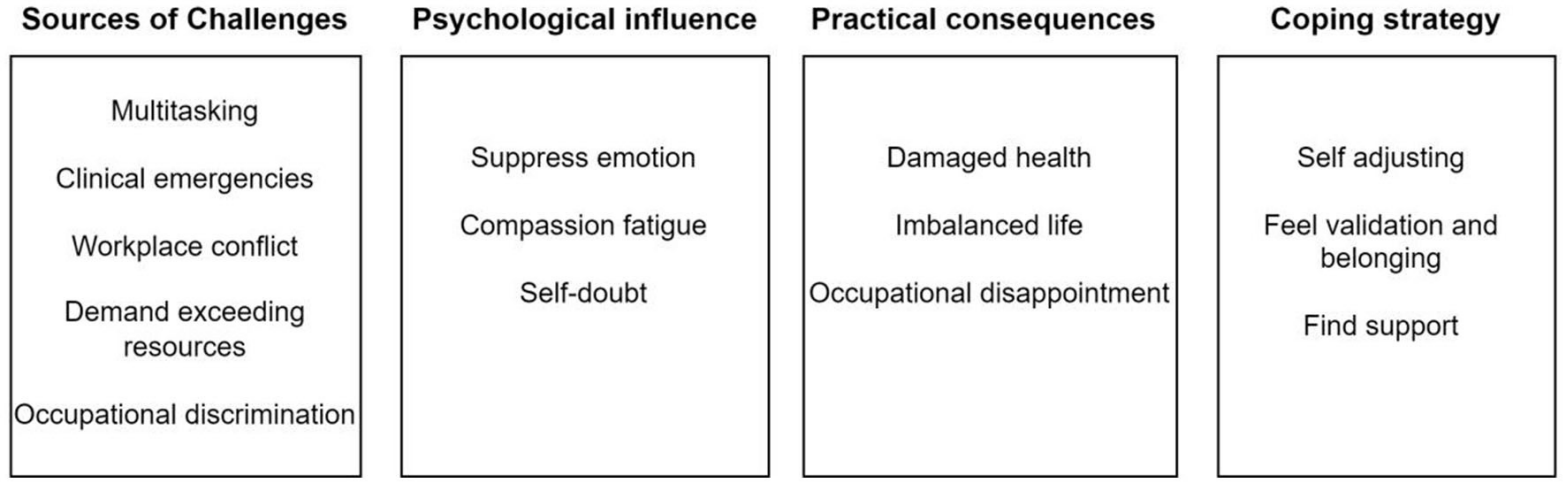
Overview of analytical and descriptive findings.

The analysis included 15 studies (39–53) that covered various Chinese provinces, such as Yunnan (*n* = 1), Shandong (*n* = 2), Hubei (*n* = 1), Guangxi (*n* = 3), Chongqing (*n* = 1), Hebei (*n* = 1), Jiangsu (*n* = 1), Hunan (*n* = 1), Sichuan (*n* = 1), Fujian (*n* = 1), Tianjin (*n* = 1), and Zhejiang (*n* = 1). These studies involved a total of 109 registered nurse and 96 nursing aides. The research methods employed in these studies included phenomenological approaches (*n* = 9) and descriptive qualitative analysis (*n* = 6). The settings for these studies encompassed Nursing homes (*n* = 11), Hospital geriatric department (*n* = 1), Nursing homes and welfare (*n* = 1), community health centers (*n* = 1), Medical-nursing integrated pension institutions (*n* = 1). All studies were published after 2015 and were original articles. The quality assessment of the literature can be found in Supplementary File 4, and the summary of the included studies can be found in Supplementary File 5. From the selected studies, four major themes emerged, revealing the challenges and coping strategies faced by nursing staff engaged in long-term care work in China. These themes include sources of challenges, psychological impacts, practical consequences, and coping strategies. Additionally, these themes are further subdivided into meaningful subthemes, as illustrated in Figure 2.

## 4 Theme 1: source of challenge

The predominant theme evident across the majority of the studies addressed the diverse challenges encountered by nursing staff. Our integrated findings revealed that these challenges primarily emanated from three key aspects: (1) Heavy clinical workload, involving multitasking and responding to clinical emergencies. (2) Professional dilemmas, encompassing workplace conflicts and demands surpassing available resources. (3) Social biases, reflecting prevalent social prejudices.

### 4.1 Multitasking

For nursing staff, multitasking is one of the most common sources of stress in their daily work. Some nursing staff express that the additional workload adds pressure to their responsibilities. “*The job doesn't have a fixed pattern, and there are tasks that I shouldn't be doing, but I have to do them* (48).” Most of the time, they need to concentrate on completing their tasks while also dealing with a chaotic environment. “*I find myself handling an older adult person's urinary problem, while simultaneously hearing two other older adult people arguing, and there's another older adult person constantly nagging* (51).” For nursing staff, this was a significant challenge as they needed to strive to balance attention to cope with demands and pressures from various sources. This required them to possess the ability to switch rapidly between different tasks and handle multiple issues simultaneously, ensuring the best possible care for patients.

### 4.2 Clinical emergencies

With the aging of bodily functions and the presence of long-term, complex, and easily deteriorating diseases, inevitable emergencies arise in the process of caring for the older adult. These emergencies stem, on the one hand, from conflicts between nursing staff and the older adult or their family members. “*The temperament of the older adult is very unpredictable… they sometimes curse at us* (46).” “*Sometimes, family members don't understand and say we haven't done our job well* (45).” On the other hand, emergencies arise from unexpected events such as falls, ingestion of harmful substances, burns, and deterioration of illnesses. “*Some older adult individuals may consume disinfectant, shampoo, and cleaning products*” (43). Certainly, the sudden deterioration of health or the death of the older adult adds immense psychological pressure on nursing staff. “*I took care of one older adult person for over 2 years, and later, when they passed away, it felt like losing a family member. During that time, I didn't want to work anymore* (47).” These sudden events impose a significant burden on nursing staff, forcing them to constantly waste a considerable amount of time and energy to prevent any potential emergencies. “*We must observe and monitor at all times to prevent unexpected occurrences* (49).” Therefore, when faced with various clinical emergencies, nursing staff needed to make rapid decisions and take effective measures within a short period to avoid conflicts and ensure the safety and health of the older adult. This placed significant demands on the responsiveness and psychological resilience of nursing staff.

### 4.3 Workplace conflict

Some nursing staff believe that workplace conflicts are also a major factor contributing to their stress. Some nursing staff think that conflicts among team members increase their pressure. This may be due to conflicts related to job assignments, as described: “*Some people don't care about their work share… very selfish* (50).” At times, these conflicts can be attributed to poor management practices: “*When management assigns tasks, there is almost no explanation, no concern for the process; they only care about the results* (50).” Certainly, unreasonable work systems can also keep staff in a constant state of stress, “*Any situation with the older adult is our responsibility* (49).” In the workplace environment, nursing staff faced intricate interpersonal relationships and stress factors, often having to deal with various workplace conflicts. Besides appropriately managing relationships with colleagues and supervisors, they had to cope with additional pressure arising from uneven job assignments and improper management. These factors had serious negative impacts on the efficiency and mental health of nursing staff, leaving them feeling physically and mentally exhausted.

### 4.4 Demand exceeding resources

Nursing personnel have diverse needs, including fair compensation, reasonable working hours, autonomy, and opportunities for career development. Some nursing staff express their concern regarding inadequate salary levels, which barely cover their daily living expenses. “*My monthly salary is only three or four thousand yuan. It's not enough to raise children and pay off the mortgage. I have no savings in recent years, and the economic pressure is great* (39).” Additionally, some nursing staff describe the challenges of long working hours and lack of sufficient rest. “*Every other day, I have to work an extra shift, from 07:00 to 19:00* (46).” Moreover, discussions among nursing staff highlight the shortage of hospital resources and staff, leading to increased pressure. “*There's no computer, a series of equipment and instruments are missing, and the blood pressure monitor has been broken for a long time before it was replaced* (48).” Nursing personnel also express their belief that rigid management systems limit their autonomy and contribute to a heavy workload. “*I have to get approval from the leaders before I can do anything. If it's not approved, I can't carry out my work* (48).” Similarly, when organizations fail to meet the career development needs of nursing staff, it can also lead to stress. “*I hope we can have more opportunities to participate in academic conferences* (49).” According to the information above, when the demands of nursing staff exceeded the resources provided by the organization, this mismatch situation arose. Such a mismatch situation could have had negative impacts on both nursing staff and healthcare institutions. Nursing staff might have felt frustrated, helpless, and defeated, affecting their work enthusiasm and efficiency. Meanwhile, healthcare institutions might have faced issues such as a decline in the quality of care, reduced patient satisfaction, and staff turnover.

### 4.5 Occupational discrimination

Discrimination is often accompanied by negative biases and stigmatization. In the context of long-term care personnel in older adult care institutions, the majority express that social occupational discrimination causes them to feel stressed. Some nursing staff feel that their work is not recognized and respected by patients or their families. “*There were family members who said behind my back, you're so young and already working in this field. When I heard that, it didn't feel good, and I thought maybe I should do this for a couple of years and then change careers* (44)”. This may be attributed to societal biases toward nursing staff in older adult care institutions. “*Someone thought they had paid for the nursing fees, so we should do everything to satisfy them* (40)”. Moreover, a lack of emotional support from family or friends can further burden their mental wellbeing. “*And some relatives heard that I was a Nursing staff, and in the nursing home, they said that I was not as good as a nanny* (39)”. Consequently, due to the fear of discrimination, nursing staff may hesitate to disclose their profession, which exacerbates feelings of inferiority. “*I don't even tell people in my hometown what kind of work I do, otherwise, they will make fun of me* (46)”.The numerous problems caused by societal prejudices led to a lack of professional identity and self-value doubt among nursing professionals. This discrimination was not only manifested in verbal attacks but also imposed a heavy psychological burden, exerting profound effects on the job performance and career choices of nursing professionals. It adversely affected their life attitudes as well.

## 5 Theme 2: psychological influence

The second theme explained the emotional impact of “work challenges on nursing staff.” When nursing professionals confronted numerous challenges in their work, it had a series of effects on their mental health. These effects could lead to emotional suppression (anxiety, worry), becoming indifferent, and self-doubt.

### 5.1 Suppress emotion

Overall, we have found that most studies have reported various negative emotions among nursing staff. These include: feelings of anxiety about not being able to fulfill their professional nursing duties. “*Many older adult people have poor vascular conditions, and it's difficult to puncture them. I'm afraid of missing the vein with the needle* (52).” Fear of complaints from family members. “*Some family members argued with our staff and even went to the leader to make a scene, so we were under a lot of pressure* (53).” Anxiety about unexpected events. “*We are particularly worried when the older adult get sick because they will feel very uncomfortable* (45).” Those negative emotions, persisting around nursing staff for an extended period, had profound implications for their physical and mental wellbeing. For instance, feelings of anxiety could impede nursing staff's ability to concentrate, affecting their decision-making. Fears of family complaints left nursing staff feeling helpless and disheartened, while concerns about the illness led them to constantly imagine the potential suffering of the older adult.

### 5.2 Compassion fatigue

Nursing staff who continue to endure occupational stress may experience compassion fatigue, which is emotional and psychological exhaustion resulting from long-term exposure to patients' suffering, illnesses, and needs. Some nursing staff express that they may feel self-blame when they cannot help patients alleviate their suffering or provide sufficient care. For example, “*I saw him getting thinner day by day, and eventually, he couldn't eat anymore. I could only give him water. It was quite distressing* (47).” Prolonged compassion fatigue leads to emotional issues such as anxiety and fear among nursing staff. “*I felt like I lost a family member when one of the older adult I was caring for passed away* (45).” Some nursing staff may become emotionally detached from patients' suffering and needs, gradually developing compassion fatigue. “*I don't talk much (frowning)… I feel lifeless all day* (46).” Others may attempt to avoid building connections with older adult patients. “*Communicating with the older adult is just impossible now; I just hope the family members can communicate well* (51).” In summary, over the course of a long-term career, some nursing staff became increasingly fatigued and ceased to express sympathy toward patients. They gradually developed a sense of numbness to the suffering of the patients, aiming to avoid being deeply entangled in it themselves. This emotional detachment manifested as a callous attitude and neglect of patients' needs. However, such practices could result in nursing staff being unable to provide the necessary support and care.

### 5.3 Self-doubt

Self-doubt is a common experience among nursing staff, often stemming from uncertainty about their own actions and abilities. Some nursing staff doubt whether they are providing the “right” care for patients. For example, “*Sometimes, when we have to restrain the hands and feet of older adult patients, it's truly heart-wrenching* (43).” Under excessive stress, some nursing staff may question their own capabilities. “*You see others handling things so effortlessly… but when it comes to me, everything becomes chaotic, and I'm unsure of what to do first or next* (52).” And the awareness of the frustration of missing out on growth opportunities. “*We work in our current positions every day, and our professional knowledge and skills are getting* (50).” Additionally, a portion of nursing staff may start questioning their career choice, wondering why they are working in a nursing home and spending their entire day caring for the older adult. “*Why do I have to serve the older adult in a nursing home and be with them all day long* (43)?” In general, self-doubt arose from a sense of responsibility in caregiving and the challenges of reality. Nursing staff began to question their decisions and even doubted their suitability for this profession. The emotions of self-doubt not only impacted the efficiency of nursing staff but also influenced their work attitudes. Faced with stress and difficulties, they felt disheartened and helpless.

## 6 Theme 3: practical consequences

The third theme focused on exploring the potential consequences of challenges faced by nursing staff in their work. Synthesizing various research findings, it was revealed that when nursing professionals encountered challenges, it led to a series of negative impacts on their health, family, and professional perspectives.

### 6.1 Damaged health

The ongoing occupational stress has significant physical effects, primarily manifesting as various physical symptoms. Some nursing staff report that the most noticeable physical manifestations of long-term high workload are bodily pains. For example, “*Wrist pain, leg pain, and backache are quite common; I didn't have these issues before working here* (45).” Prolonged work-related stress can lead to chronic fatigue and a sense of exhaustion, leaving nursing staff feeling drained. “*When I get home, I just want to lie down, I don't feel like doing anything, I don't even want to eat* (48).” Insomnia is also a common health problem among nursing staff. “*I lose sleep almost every day* (45).” These physical symptoms were not accidental occurrences; they were more likely to be cumulative effects of long-term workloads on the body. In addition to surface pain and fatigue, occupational stress could also trigger physiological changes within the body. Research indicated (54) that prolonged work stress could reduce the functionality of the immune system, thereby increasing the risk of illness.

### 6.2 Imbalanced life

The lack of work-life balance leaves nursing staff feeling that their lives revolve around their work. Some nursing staff report that they have minimal vacation time and even less time for rest, which has a negative impact on their personal lives. For instance, “*We work almost 12 h a day… get 1 day off per week. We can barely go back home or go out* (45).” For some nursing staff, they have little to no time for socializing because work takes precedence over any significant personal life activities. “*I don't even have time to take my son to the park (cries)* (48).” When nursing staff face challenges in their personal lives, such as family illnesses, the inflexibility of their work schedules adds to their stress. “*One time, my dad got sick, and (the director) wouldn't grant me leave, saying that taking leave would*. *mean a deduction in my salary* (46).” In general, the lack of balance between work and life has profound effects on the lives of nursing staff. This situation is evident not only in the absence of holidays and rest time but also in their inability to engage in essential activities in their personal lives, such as spending time with family, socializing, and leisure. This circumstance may lead to a sense of neglect among the family and friends of nursing staff, triggering a series of family and social conflicts. However, due to the intensity and scheduling demands of their work, it is nearly impossible for personal life to coexist with it.

### 6.3 Occupational disappointment

The ongoing stress gradually leads nursing staff to question their chosen profession. This pressure may arise from misconceptions they hold about the field of older adult care. “*When I first started working in the geriatric ward, I felt quite uneasy witnessing nursing staff feeding and bathing the older adult. It created a significant psychological gap* (53).” Additionally, the pressure could be influenced by comparisons among peers. “*My classmates earn almost twice as much money as I do in general hospitals, which makes me reluctant to continue* (39).” As the pressure continues to build, some nursing staff express a growing sense of dissatisfaction in their career. “*To be honest, I don't believe long-term nursing staff play a vital role in the process of older adult care… It seems to revolve around injections, infusions, and medications* (39).” *Excessive pressure can even impact the mental health of nursing staff. “I find myself losing hope in life”* (48). Disappointment may serve as the motivation for nursing staff to consider early resignation or explore alternative job opportunities. “*This job is too challenging… I think I want to quit* (47).” This poses a challenge not only for individual nursing staff but also for the entire older adult care industry. The nature of older adult care work, distinct from clinical tasks, demands nursing staff to invest more emotion and care. Regrettably, the wages of Chinese older adult care personnel are notably lower compared to other nursing professions. This economic pressure undoubtedly diminishes their enthusiasm and dedication to the older adult care profession.

## 7 Theme 4: coping strategy

The fourth theme elaborated on the coping strategies adopted by nursing staff when they faced challenges in their work. The research findings indicated that nursing staff took a series of measures to address these challenges, primarily encompassing self-regulation, establishing identity and a sense of belonging, and seeking support. The effective application of these strategies contributed to nursing staff better addressing the challenges encountered in their work, thereby enhancing work efficiency and quality.

### 7.1 Self-adjusting

Employee self-care is considered helpful in relieving stress. Some nursing staff have expressed that they alleviate stress by going for a walk or a run, saying, “*Going out for a run, clearing my mind, and coming back, I realize that things aren't so bad after all* (43).” Some nursing staff also believe that understanding the reasons behind conflicts in the older adult can help them avoid stress, saying, “*He didn't intentionally hit or scold me; he's just sick and can't control himself* (42).” Additionally, some nursing staff have mentioned that accepting reality through self-comfort and encouragement is an inevitable but effective way to relieve stress, saying, “*There's no other choice but to persevere, and eventually, I slowly come to accept it* (44).” In general, employee self-regulation is not merely a singular approach but a composite of diverse strategies. Through physical exercise, mutual understanding, and a rational awareness of reality, nursing staff can better cope with various challenges in their work and maintain a positive and proactive state.

### 7.2 Feel validation and belonging

Helping nursing staff gain recognition and a sense of belonging is one of the important ways to alleviate work stress. Some nursing staff express that when patients or management can respect and recognize them, and support their work, they feel more motivated and satisfied. “*I am touched to receive everyone's recognition. It's great!*” (41). Another group of nursing staff mentions that in the process of long-term care for the older adult, being able to establish connections beyond the caregiver-patient relationship makes them feel a sense of “home.” “*I am happy, and the older adult treat me well, just like my parents. I also like them* (40).” Overall, nursing staff, through the establishment of deep interpersonal relationships in their work, can gain recognition and a sense of belonging, which has a positive impact on both their psychological wellbeing and job effectiveness. This emotional connection aids nursing staff in fulfilling their duties more effectively and finding emotional support in high-pressure work environments, thereby fostering greater cohesion within the entire medical team.

### 7.3 Find support

When facing stress, nursing staff may seek support from others to help them overcome difficulties. Emotional support from managers, colleagues, and family members can help nursing staff enhance their ability to cope with stress. Some nursing staff have expressed that they receive excellent teamwork support from their colleagues, which helps them deal with the challenges they encounter at work. “*Everyone takes care of me, covering my shifts during lunchtime and allowing me to leave early in the evening. During my pregnancy, I wasn't burdened with additional tasks* (41).” Some nursing staff have mentioned receiving material support from their managers, which eases their workload. “*The director bought auxiliary tools to lighten our workload* (46).” Additionally, some nursing staff have mentioned receiving emotional support from their family or friends outside of work, which assists them in dealing with stress. “*My mother and husband have provided me with the greatest support* (42).” Therefore, when nursing staff face pressure, they often seek external support to help them cope with the challenges of their work. In addition to material support and the care of management, emotional support from colleagues and family members is also crucial. This support can assist nursing staff in better addressing the challenges of their work, enhancing their confidence and courage to face future obstacles.

## 8 Discussion

This study reviewed 15 qualitative research studies, exploring the challenges faced by nursing staff in Chinese long-term care institutions and their coping strategies. Relevant information was gathered by searching databases. The results emphasize challenges encountered by nursing staff in their professional roles, including the complexity of clinical responsibilities, unforeseeable events, scarcity of resources, concurrent demands, workplace conflicts, and societal biases. These challenges adversely affect the psychological wellbeing of nursing professionals, manifested as emotional suppression, indifference, and self-doubt. In the dual context of psychological stress and work challenges, the health, quality of life, and job satisfaction of nursing staff are further compromised. Nursing personnel employ various coping strategies, managing work pressure and emotional distress through self-regulation, and actively seeking support for recognition and a sense of belonging.

Our findings highlight the challenges faced by nursing staff in long-term care facilities in China. These challenges can be broadly categorized into clinical, workplace, and social aspects. Addressing challenges in clinical and workplace settings may be relatively easier compared to those in the social domain. Therefore, to tackle the challenges faced by nursing staff in clinical and workplace settings, managers should make efforts in at least two areas. (1) Implement effective management strategies. Managers should formulate reasonable human resource plans and establish scientific workflows based on workload and staffing ratios (55). This helps prevent negative psychological states caused by excessive workloads or unfair treatment (56). (2) Provide resource support. Our results indicate a shortage of material, emotional, and informational resources for nursing staff. This implies that managers should establish corresponding support systems to address resource deficiencies. For instance, in terms of material resources, it is essential not only to provide an adequate supply of resources but also to ensure timely updates and replenishments. Regarding emotional resources, managers need to create a positive work environment (57), offer psychological counseling (58), or adopt more humane management approaches. Similarly, organizations should provide relevant training and learning opportunities (59), offer promotion channels (56), and stay informed about the latest medical information and technology (AI) (60) to meet nursing staff' knowledge and skill needs.

We identified that occupational discrimination poses significant challenges to the psychological wellbeing of long-term nursing staff in China, and this issue was closely intertwined with their social status. Our findings reveal that the social status of nursing staff in Chinese nursing homes is comparatively low, leading to feelings of being undervalued and lacking respect (61). A specific study underscores that discrimination was not inherent to the nature of the job itself but rather emanates from the societal label attached to “nursing home” in Chinese culture (62). On one hand, the combination of low wages and substantial workloads might have contributed to the societal undervaluation of long-term care work. On the other hand, this phenomenon could be attributed to the emphasis on the traditional Chinese cultural value of “family-based care for the older adult.” Placing parents in nursing homes might be perceived by some as an act of filial impiety, thereby fostering a degree of discrimination against such institutions.

This investigation revealed a noteworthy correlation between elevated stress levels and a considerable impact on the work-life equilibrium of nursing personnel. These findings align with established evidence in the literature, particularly highlighting a robust association between an onerous workload and heightened work-related stress. Notably, a study (63) demonstrated that an increased workload and limited personal time were key contributors to work-related stress. Intriguingly, the imbalance in work-life dynamics among nursing staff within Chinese long-term care facilities exerted more pronounced adverse effects on their families. This phenomenon could potentially be elucidated by the influence of traditional Chinese gender roles, where women frequently shoulder additional family responsibilities, such as childcare or eldercare (19). Contrarily, our research indicated that nursing staff in long-term care facilities encounter limited opportunities to fulfill their familial duties due to extended working hours.

Our investigation also revealed that nursing staff employed diverse coping strategies when confronted with challenges, including cognitive restructuring and seeking support. This information held implications for the formulation of intervention measures. For instance, institutions could have offered psychological support to nursing staff through avenues such as counseling or meditation training (64). Alternatively, emotional support can be fostered by providing social activities to encourage the development of interpersonal relationships. Notably, prior research had demonstrated the efficacy of mindfulness therapy in mitigating stress levels among nursing staff (25). Likewise, institutions might have considered alleviating the burden on nursing staff by reducing their working hours and workload (64), ensuring they had ample time and energy to sustain social relationships and mitigate stress arising from familial or peer interactions.

## 9 Conclusion

This study provides a detailed examination of the challenges confronting nursing staff within long-term care institutions in China and explores their coping experiences. The encountered challenges are diverse, exerting adverse effects on the psychological wellbeing of nursing staff and resulting in unfavorable consequences in areas such as health, life, and career. Various coping strategies can be employed by nursing staff to address these work-related challenges. It is imperative for organizations, managers, and mental health experts to collaborate in implementing more efficacious intervention measures, fostering the advancement of nursing staff toward more favorable coping strategies.

## 10 Strength and limitations

This study, through the method of qualitative meta-analysis, collected and organized the challenges faced by nursing staff in Chinese long-term care institutions and their coping experiences. Some quantitative systematic reviews discussed the challenges faced by nursing staff in Chinese long-term care institutions (65) (nursing time, financial resources, role and personal stress, preparedness, social roles, and lack of sufficient formal support). Although we know about “those” challenges, it is still unclear “why” these challenges affect nursing staff and “how” nursing staff cope with these challenges. Therefore, the essence of this study lies in integrating fragmented information from qualitative research into a more consistent and comprehensive theoretical framework, providing new perspectives and understanding for the development of this field.

This study employed meticulous search strategies to facilitate systematic retrieval. However, it is crucial to acknowledge that, in adherence to the inclusion criteria, quantitative studies, non-English and non-Chinese papers, and specific gray literature were excluded. Such exclusions may introduce information bias. Secondly, the study utilized the JBI quality assessment tool, primarily addressing criteria related to study design, methodology, data analysis, and other factors influencing the validity of study results. Nonetheless, it may not encompass the quality assessment of detailed descriptions in the report concerning the study's purpose, background, and sample characteristics. Therefore, the assessment of data quality in these aspects within the report remains unaddressed. Thirdly, the study employed the CERQual method to evaluate the confidence in the review results. During this process, eight out of 14 results were accorded high confidence, while six out of 14 received moderate confidence. This outcome may be attributed to the limited number of primary studies (participants) contributing to the review and the heterogeneity of the samples, both of which impact the credibility of the results.

## Data availability statement

The original contributions presented in the study are included in the article/Supplementary material, further inquiries can be directed to the corresponding author.

## Author contributions

SZ: Writing—original draft, Writing—review & editing. XX: Writing—original draft, Writing—review & editing. YA: Writing—original draft, Writing—review & editing. AZ: Writing—original draft. CZ: Writing—original draft. HH: Writing—review & editing. YW: Writing—review & editing.
