# Supplementary material for: Challenges and coping experiences faced by nursing staff in long-term care facilities in China: a qualitative meta-analysis
Source: Front Public Health. 2024 Jan 8;11:1302481. doi: 10.3389/fpubh.2023.1302481 (PMC10800415; doi:10.3389/fpubh.2023.1302481)
Supplement: Supplementary file 1 [file Table_1.docx]

(1) Nurse, nurse practitioners, Caregivers, Nursing assistants, Nurse*, Nursing staff*, Nursing, health personnel*, health professional*, healthcare professional*, health worker*, healthcare worker*, health staff, healthcare staff, health practitioner*, healthcare practitioner*, Nurse Practitioner, Practitioner*, Nurse, Staff, Nursing, Staffs, Nursing, Personnel, Nursing, Nursing Personnel, Registered Nurses, Nurse, Registered, Nurses, Registered, Registered Nurse, Caregiver, Carers, Carer, Care Givers, Care Giver, Spouse Caregivers, Caregiver, Spouse, Caregivers, Spouse, Spouse Caregiver, Family Caregivers, Caregiver, Family, Caregivers, Family, Family Caregiver, Informal Caregivers, Caregiver, Informal, Caregivers, Informal, Informal Caregiver, Assistant*, Nursing, Nursing Assistant, Nurses' Aides, Aides, Nurses', Nurse's Aides, Nurses Aides, Nurses' Aide, Nursing Auxiliaries, Auxiliaries, Nursing, Auxiliary, Nursing, Nursing Auxiliary, Nurse Aide, Aide, Nurse, Aides, Nurse, Nurse Aides

(2) long-term care, nursing home, Adult Day Care Centers, Long term care*, Long-term care*, care, long-term, nursing home*, residential care*, residential home*, care home*, residential facilit*, old age home*, long term facilit*, assisted living facilit*, Day Care Center*, Adult, Adult Day Care Center

(3) stress, psychological, pressure, Psychological Stresses, Stresses, Psychological, Stress, Psychologic, Psychologic Stress, Stressor, Psychological, Psychological Stressor, Psychological Stressors, Stressors, Psychological, Psychological Stress

(4) challenge, Barrier*, inhibitor*, obstacle*, hinder*, challeng*, limit*, impediment*, difficult*, problem*

(5) Adaptation, psychological, Adaptation, Psychologic, Psychologic Adaptation, Psychological Adaptation, Adjustment, Coping Behavior, Behavior, Coping, Behaviors, Coping, Coping Behaviors, Coping Skills, Coping Skill, Skill, Coping, Skills, Coping, Coping Strategies, Coping Strategy, Strategies, Coping, Strategy, Coping, Behavior, Adaptive, Adaptive Behavior, Adaptive Behaviors, Behaviors, Adaptive

(6) Experience, Feeling, Perception, Perspective, Emotion, Attitude, Response, Respond*, Cop*, Manage*, Involve*, Engag*, Participat*

(7) Qualitative research, research, semi-structured, semistructured, unstructured, informal, in-depth, indepth, face-to-face, structured guide, guides interview*, discussion*, questionnaire*, focus group*, qualitative, ethnograph*, fieldwork, field work, key informant, interviews as topic, focus group narration, personal narratives as topic, theme, thematic, ethnological research, phenomenol*, grounded theory, grounded study, grounded studies, grounded research, grounded analysis, grounded analyses, life story, life stories, emic, etic, hermeneutics, heuristic*, semiotic, data saturation, participant observation, action research, cooperative inquiry, co-operative inquiry, field study, field studies, field research, theoretical sample, theoretical samples, theoretical sampling, purposive sampling, purposive sample*, lived experience*, content analysis, content analyses, discourse, narrative analysis, narrative analyses, heidegger*, colaizzi, spiegelberg, van manen*, van kaam, merleau ponty, husserl*, Foucault, Corbin, Strauss, Glaser

(8) China, People's Republic of China, Mainland China, Sinkiang, Inner Mongolia, Manchuria

| Steps | Search strategy |
| --- | --- |
| **Pubmed** | |
| #1 | (((((((((((((((((((((((((((((((((((((((((((((((((((((((((Nurse[MeSH Terms]) OR (nurse practitioners[MeSH Terms])) OR (Caregivers[MeSH Terms])) OR (Nursing assistants[MeSH Terms])) OR (Nurse*)) OR (Nursing staff*)) OR (Nursing)) OR (health personnel*)) OR (health professional*)) OR (healthcare professional*)) OR (health worker*)) OR (healthcare worker*)) OR (health staff)) OR (healthcare staff)) OR (health practitioner*)) OR (healthcare practitioner*)) OR (Nurse Practitioner)) OR (Practitioner*, Nurse)) OR (Staff, Nursing)) OR (Staffs, Nursing)) OR (Personnel, Nursing)) OR (Nursing Personnel)) OR (Registered Nurses)) OR (Nurse, Registered)) OR (Nurses, Registered)) OR (Registered Nurse)) OR (Caregiver)) OR (Carers)) OR (Carer)) OR (Care Givers)) OR (Care Giver)) OR (Spouse Caregivers)) OR (Caregiver, Spouse)) OR (Caregivers, Spouse)) OR (Spouse Caregiver)) OR (Family Caregivers)) OR (Caregiver, Family)) OR (Caregivers, Family)) OR (Family Caregiver)) OR (Informal Caregivers)) OR (Caregiver, Informal)) OR (Caregivers, Informal)) OR (Informal Caregiver)) OR (Assistant*, Nursing)) OR (Nursing Assistant)) OR (Nurses' Aides)) OR (Aides, Nurses')) OR (Nurse's Aides)) OR (Nurses Aides)) OR (Nurses' Aide)) OR (Nursing Auxiliaries)) OR (Auxiliaries, Nursing)) OR (Auxiliary, Nursing)) OR (Nursing Auxiliary)) OR (Nurse Aide)) OR (Aide, Nurse)) OR (Aides, Nurse)) OR (Nurse Aides) |
| #2 | (((((((((((((((long-term care[MeSH Terms]) OR (nursing home[MeSH Terms])) OR (Adult Day Care Centers[MeSH Terms])) OR (Long term care*)) OR (Long-term care*)) OR (care, long-term)) OR (nursing home*)) OR (residential care*)) OR (residential home*)) OR (care home*)) OR (residential facilit*)) OR (old age home*)) OR (long term facilit*)) OR (assisted living facilit*)) OR (Day Care Center*, Adult)) OR (Adult Day Care Center) |
| #3 | ((((((((((stress, psychological[MeSH Terms]) OR (pressure[MeSH Terms])) OR (Psychological Stresses)) OR (Stresses, Psychological)) OR (Stress, Psychologic)) OR (Psychologic Stress)) OR (Stressor, Psychological)) OR (Psychological Stressor)) OR (Psychological Stressors)) OR (Stressors, Psychological)) OR (Psychological Stress) |
| #4 | (((((((((challenge) OR (Barrier*)) OR (inhibitor*)) OR (obstacle*)) OR (hinder*)) OR (challeng*)) OR (limit*)) OR (impediment*)) OR (difficult*)) OR (problem*) |
| #5 | ((((((((((((((((((((Adaptation, psychological[MeSH Terms]) OR (Adaptation, Psychologic)) OR (Psychologic Adaptation)) OR (Psychological Adaptation)) OR (Adjustment)) OR (Coping Behavior)) OR (Behavior, Coping)) OR (Behaviors, Coping)) OR (Coping Behaviors)) OR (Coping Skills)) OR (Coping Skill)) OR (Skill, Coping)) OR (Skills, Coping)) OR (Coping Strategies)) OR (Coping Strategy)) OR (Strategies, Coping)) OR (Strategy, Coping)) OR (Behavior, Adaptive)) OR (Adaptive Behavior)) OR (Adaptive Behaviors)) OR (Behaviors, Adaptive) |
| #6 | ((((((((((((Experience) OR (Feeling)) OR (Perception)) OR (Perspective)) OR (Emotion)) OR (Attitude)) OR (Response)) OR (Respond*)) OR (Cop*)) OR (Manage*)) OR (Involve*)) OR (Engag*)) OR (Participat*) |
| #7 | (((((((((((((((((((((((((((((((((((((((((((((((((((((((((((((((((Qualitative research[MeSH Terms]) OR (research[MeSH Terms]) OR (semi-structured)) OR (semistructured)) OR (unstructured)) OR (informal)) OR (in-depth)) OR (indepth)) OR (face-to-face)) OR (structured guide)) OR (guides interview*)) OR (discussion*)) OR (questionnaire*)) OR (focus group*)) OR (qualitative)) OR (ethnograph*)) OR (fieldwork)) OR (field work)) OR (key informant)) OR (interviews as topic)) OR (focus group narration)) OR (personal narratives as topic)) OR (theme)) OR (thematic)) OR (ethnological research)) OR (phenomenol*)) OR (grounded theory)) OR (grounded study)) OR (grounded studies)) OR (grounded research)) OR (grounded analysis)) OR (grounded analyses)) OR (life story)) OR (life stories)) OR (emic)) OR (etic)) OR (hermeneutics)) OR (heuristic*)) OR (semiotic)) OR (data saturation)) OR (participant observation)) OR (action research)) OR (cooperative inquiry)) OR (co-operative inquiry)) OR (field study)) OR (field studies)) OR (field research)) OR (theoretical sample)) OR (theoretical samples)) OR (theoretical sampling)) OR (purposive sampling)) OR (purposive sample*)) OR (lived experience*)) OR (content analysis)) OR (content analyses)) OR (discourse)) OR (narrative analysis)) OR (narrative analyses)) OR (heidegger*)) OR (colaizzi)) OR (spiegelberg)) OR (van manen*)) OR (van kaam)) OR (merleau ponty)) OR (husserl*)) OR (Foucault or Corbin)) OR (Strauss or Glaser) |
| #8 | (((((China[MeSH Terms]) OR (People's Republic of China)) OR (Mainland China)) OR (Sinkiang)) OR (Inner Mongolia)) OR (Manchuria) |
| #9 | #3 OR #4 |
| #10 | #5 OR #6 |
|  | #1 AND #2 AND #9 AND #10 #AND #7 AND #8 |
| **Web of science** | |
| #1 | (((((((((((((((((((((((((((((((((((((((((((((((((((((((((TS=(Nurse)) OR TS=(nurse practitioners)) OR TS=(Caregivers[MeSH Terms])) OR TS=(Nursing assistants[MeSH Terms])) OR TS=(Nurse*)) OR TS=(Nursing staff*)) OR TS=(Nursing)) OR TS=(health personnel*)) OR TS=(health professional*)) OR TS=(healthcare professional*)) OR TS=(health worker*)) OR TS=(healthcare worker*)) OR TS=(health staff)) OR TS=(healthcare staff)) OR TS=(health practitioner*)) OR TS=(healthcare practitioner*)) OR TS=(Nurse Practitioner)) OR TS=(Practitioner*, Nurse)) OR TS=(Staff, Nursing)) OR TS=(Staffs, Nursing)) OR TS=(Personnel, Nursing)) OR TS=(Nursing Personnel)) OR TS=(Registered Nurses)) OR TS=(Nurse, Registered)) OR TS=(Nurses, Registered)) OR TS=(Registered Nurse)) OR TS=(Caregiver)) OR TS=(Carers)) OR TS=(Carer)) OR TS=(Care Givers)) OR TS=(Care Giver)) OR TS=(Spouse Caregivers)) OR TS=(Caregiver, Spouse)) OR TS=(Caregivers, Spouse)) OR TS=(Spouse Caregiver)) OR TS=(Family Caregivers)) OR TS=(Caregiver, Family)) OR TS=(Caregivers, Family)) OR TS=(Family Caregiver)) OR TS=(Informal Caregivers)) OR TS=(Caregiver, Informal)) OR TS=(Caregivers, Informal)) OR TS=(Informal Caregiver)) OR TS=(Assistant*, Nursing)) OR TS=(Nursing Assistant)) OR TS=(Nurses' Aides)) OR TS=(Aides, Nurses')) OR TS=(Nurse's Aides)) OR TS=(Nurses Aides)) OR TS=(Nurses' Aide)) OR TS=(Nursing Auxiliaries)) OR TS=(Auxiliaries, Nursing)) OR ALL=(Auxiliary, Nursing)) OR ALL=(Nursing Auxiliary)) OR ALL=(Nurse Aide)) OR ALL=(Aide, Nurse)) OR ALL=(Aides, Nurse)) OR ALL=(Nurse Aides) |
| #2 | (((((((((((((((TS=(long-term care)) OR TS=(nursing home)) OR TS=(Adult Day Care Centers)) OR TS=(Long term care*)) OR TS=(Long-term care*)) OR TS=(care, long-term)) OR TS=(nursing home*)) OR TS=(residential care*)) OR TS=(residential home*)) OR TS=(care home*)) OR TS=(residential facilit*)) OR TS=(old age home*)) OR TS=(long term facilit*)) OR TS=(assisted living facilit*)) OR TS=(Day Care Center*, Adult)) OR TS=(Adult Day Care Center) |
| #3 | ((((((((((TS=(stress, psychological)) OR TS=(pressure)) OR TS=(Psychological Stresses)) OR TS=(Stresses, Psychological)) OR TS=(Stress, Psychologic)) OR TS=(Psychologic Stress)) OR TS=(Stressor, Psychological)) OR TS=(Psychological Stressor)) OR TS=(Psychological Stressors)) OR TS=(Stressors, Psychological)) OR TS=(Psychological Stress) |
| #4 | (((((((((TS=(challenge)) OR TS=(Barrier*)) OR TS=(inhibitor*)) OR TS=(obstacle*)) OR TS=(hinder*)) OR TS=(challeng*)) OR TS=(limit*)) OR TS=(impediment*)) OR TS=(difficult*)) OR TS=(problem*) |
| #5 | ((((((((((((((((((((TS=(Adaptation, psychological)) OR TS=(Adaptation, Psychologic)) OR TS=(Psychologic Adaptation)) OR TS=(Psychological Adaptation)) OR TS=(Adjustment)) OR TS=(Coping Behavior)) OR TS=(Behavior, Coping)) OR TS=(Behaviors, Coping)) OR TS=(Coping Behaviors)) OR TS=(Coping Skills)) OR TS=(Coping Skill)) OR TS=(Skill, Coping)) OR TS=(Skills, Coping)) OR TS=(Coping Strategies)) OR TS=(Coping Strategy)) OR TS=(Strategies, Coping)) OR TS=(Strategy, Coping)) OR TS=(Behavior, Adaptive)) OR TS=(Adaptive Behavior)) OR TS=(Adaptive Behaviors)) OR TS=(Behaviors, Adaptive) |
| #6 | ((((((((((((TS=(Experience)) OR TS=(Feeling)) OR TS=(Perception)) OR TS=(Perspective)) OR TS=(Emotion)) OR TS=(Attitude)) OR TS=(Response)) OR TS=(Respond*)) OR TS=(Cop*)) OR TS=(Manage*)) OR TS=(Involve*)) OR TS=(Engag*)) OR TS=(Participat*) |
| #7 | (((((((((((((((((((((((((((((((((((((((((((((((((((((((((((((((((TS=(Qualitative research)) OR TS=(research) OR TS=(semi-structured)) OR TS=(semistructured)) OR TS=(unstructured)) OR TS=(informal)) OR TS=(in-depth)) OR TS=(indepth)) OR TS=(face-to-face)) OR TS=(structured guide)) OR TS=(guides interview*)) OR TS=(discussion*)) OR TS=(questionnaire*)) OR TS=(focus group*)) OR TS=(qualitative)) OR TS=(ethnograph*)) OR TS=(fieldwork)) OR TS=(field work)) OR TS=(key informant)) OR TS=(interviews as topic)) OR TS=(focus group narration)) OR TS=(personal narratives as topic)) OR TS=(theme)) OR TS=(thematic)) OR TS=(ethnological research)) OR TS=(phenomenol*)) OR TS=(grounded theory)) OR TS=(grounded study)) OR TS=(grounded studies)) OR TS=(grounded research)) OR TS=(grounded analysis)) OR TS=(grounded analyses)) OR TS=(life story)) OR TS=(life stories)) OR TS=(emic)) OR TS=(etic)) OR TS=(hermeneutics)) OR TS=(heuristic*)) OR TS=(semiotic)) OR TS=(data saturation)) OR TS=(participant observation)) OR TS=(action research)) OR TS=(cooperative inquiry)) OR TS=(co-operative inquiry)) OR TS=(field study)) OR TS=(field studies)) OR TS=(field research)) OR TS=(theoretical sample)) OR TS=(theoretical samples)) OR TS=(theoretical sampling)) OR TS=(purposive sampling)) OR TS=(purposive sample*)) OR TS=(lived experience*)) OR TS=(content analysis)) OR TS=(content analyses)) OR TS=(discourse)) OR TS=(narrative analysis)) OR TS=(narrative analyses)) OR TS=(heidegger*)) OR TS=(colaizzi)) OR TS=(spiegelberg)) OR TS=(van manen*)) OR TS=(van kaam)) OR TS=(merleau ponty)) OR TS=(husserl*)) OR TS=(Foucault or Corbin)) OR TS=(Strauss or Glaser) |
| #8 | (((((TS=(China)) OR TS=(People's Republic of China)) OR TS=(Mainland China)) OR TS=(Sinkiang)) OR TS=(Inner Mongolia)) OR TS=(Manchuria) |
| #9 | #3 OR #4 |
| #10 | #5 OR #6 |
|  | #1 AND #2 AND #9 AND #10 #AND #7 AND #8 |
| **Cochrane Library** | |
| #1 | (Nurse):ti,ab,kw OR (nurse practitioners):ti,ab,kw OR (Caregivers):ti,ab,kw OR (Nursing assistants):ti,ab,kw OR (Nurse*):ti,ab,kw OR (Nursing staff*):ti,ab,kw OR (Nursing):ti,ab,kw OR (health personnel*):ti,ab,kw OR (health professional*):ti,ab,kw OR (healthcare professional*):ti,ab,kw OR (health worker*):ti,ab,kw OR (healthcare worker*):ti,ab,kw OR (health staff):ti,ab,kw OR (healthcare staff):ti,ab,kw OR (health practitioner*):ti,ab,kw OR (healthcare practitioner*):ti,ab,kw OR (Nurse Practitioner):ti,ab,kw OR (Practitioner*, Nurse):ti,ab,kw OR (Staff, Nursing):ti,ab,kw OR (Staffs, Nursing):ti,ab,kw OR (Personnel, Nursing):ti,ab,kw OR (Nursing Personnel):ti,ab,kw OR (Registered Nurses):ti,ab,kw OR (Nurse, Registered):ti,ab,kw OR (Nurses, Registered):ti,ab,kw OR (Registered Nurse):ti,ab,kw OR (Caregiver):ti,ab,kw OR (Carers):ti,ab,kw OR (Carer):ti,ab,kw OR (Care Givers):ti,ab,kw OR (Care Giver):ti,ab,kw OR (Spouse Caregivers):ti,ab,kw OR (Caregiver, Spouse):ti,ab,kw OR (Caregivers, Spouse):ti,ab,kw OR (Spouse Caregiver):ti,ab,kw OR (Family Caregivers):ti,ab,kw OR (Caregiver, Family):ti,ab,kw OR (Caregivers, Family):ti,ab,kw OR (Family Caregiver):ti,ab,kw OR (Informal Caregivers):ti,ab,kw OR (Caregiver, Informal):ti,ab,kw OR (Caregivers, Informal):ti,ab,kw OR (Informal Caregiver):ti,ab,kw OR (Assistant*, Nursing):ti,ab,kw OR (Nursing Assistant):ti,ab,kw OR (Nurses' Aides):ti,ab,kw OR (Aides, Nurses'):ti,ab,kw OR (Nurse's Aides):ti,ab,kw OR (Nurses Aides):ti,ab,kw OR (Nurses' Aide):ti,ab,kw OR (Nursing Auxiliaries):ti,ab,kw OR (Auxiliaries, Nursing):ti,ab,kw OR (Auxiliary, Nursing):ti,ab,kw OR (Nursing Auxiliary):ti,ab,kw OR (Nurse Aide):ti,ab,kw OR (Aide, Nurse):ti,ab,kw OR (Aides, Nurse):ti,ab,kw OR (Nurse Aides):ti,ab,kw |
| #2 | (long-term care):ti,ab,kw OR (nursing home):ti,ab,kw OR (Adult Day Care Centers):ti,ab,kw OR (Long term care*):ti,ab,kw OR (Long-term care*):ti,ab,kw OR (care, long-term):ti,ab,kw OR (nursing home*):ti,ab,kw OR (residential care*):ti,ab,kw OR (residential home*):ti,ab,kw OR (care home*):ti,ab,kw OR (residential facilit*):ti,ab,kw OR (old age home*):ti,ab,kw OR (long term facilit*):ti,ab,kw OR (assisted living facilit*):ti,ab,kw OR (Day Care Center*, Adult):ti,ab,kw OR (Adult Day Care Center):ti,ab,kw |
| #3 | (stress, psychological):ti,ab,kw OR (pressure):ti,ab,kw OR (Psychological Stresses):ti,ab,kw OR (Stresses, Psychological):ti,ab,kw OR (Stress, Psychologic):ti,ab,kw OR (Psychologic Stress):ti,ab,kw OR (Stressor, Psychological):ti,ab,kw OR (Psychological Stressor):ti,ab,kw OR (Psychological Stressors):ti,ab,kw OR (Stressors, Psychological):ti,ab,kw OR (Psychological Stress):ti,ab,kw |
| #4 | (challenge):ti,ab,kw OR (Barrier*):ti,ab,kw OR (inhibitor*):ti,ab,kw OR (obstacle*):ti,ab,kw OR (hinder*):ti,ab,kw OR (challeng*):ti,ab,kw OR (limit*):ti,ab,kw OR (impediment*):ti,ab,kw OR (difficult*):ti,ab,kw OR (problem*):ti,ab,kw |
| #5 | (Adaptation, psychological):ti,ab,kw OR (Adaptation, Psychologic):ti,ab,kw OR (Psychologic Adaptation):ti,ab,kw OR (Psychological Adaptation):ti,ab,kw OR (Adjustment):ti,ab,kw OR (Coping Behavior):ti,ab,kw OR (Behavior, Coping):ti,ab,kw OR (Behaviors, Coping):ti,ab,kw OR (Coping Behaviors):ti,ab,kw OR (Coping Skills):ti,ab,kw OR (Coping Skill):ti,ab,kw OR (Skill, Coping):ti,ab,kw OR (Skills, Coping):ti,ab,kw OR (Coping Strategies):ti,ab,kw OR (Coping Strategy):ti,ab,kw OR (Strategies, Coping):ti,ab,kw OR (Strategy, Coping):ti,ab,kw OR (Behavior, Adaptive):ti,ab,kw OR (Adaptive Behavior):ti,ab,kw OR (Adaptive Behaviors):ti,ab,kw OR (Behaviors, Adaptive):ti,ab,kw |
| #6 | (Experience):ti,ab,kw OR (Feeling):ti,ab,kw OR (Perception):ti,ab,kw OR (Perspective):ti,ab,kw OR (Emotion):ti,ab,kw OR (Attitude):ti,ab,kw OR (Response):ti,ab,kw OR (Respond*):ti,ab,kw OR (Cop*):ti,ab,kw OR (Manage*):ti,ab,kw OR (Involve*):ti,ab,kw OR (Engag*):ti,ab,kw OR (Participat*):ti,ab,kw |
| #7 | (Qualitative research):ti,ab,kw OR (research) OR (semi-structured):ti,ab,kw OR (semistructured):ti,ab,kw OR (unstructured):ti,ab,kw OR (informal):ti,ab,kw OR (in-depth):ti,ab,kw OR (indepth):ti,ab,kw OR (face-to-face):ti,ab,kw OR (structured guide):ti,ab,kw OR (guides interview*):ti,ab,kw OR (discussion*):ti,ab,kw OR (questionnaire*):ti,ab,kw OR (focus group*):ti,ab,kw OR (qualitative):ti,ab,kw OR (ethnograph*):ti,ab,kw OR (fieldwork):ti,ab,kw OR (field work):ti,ab,kw OR (key informant):ti,ab,kw OR (interviews as topic):ti,ab,kw OR (focus group narration):ti,ab,kw OR (personal narratives as topic):ti,ab,kw OR (theme):ti,ab,kw OR (thematic):ti,ab,kw OR (ethnological research):ti,ab,kw OR (phenomenol*):ti,ab,kw OR (grounded theory):ti,ab,kw OR (grounded study):ti,ab,kw OR (grounded studies):ti,ab,kw OR (grounded research):ti,ab,kw OR (grounded analysis):ti,ab,kw OR (grounded analyses):ti,ab,kw OR (life story):ti,ab,kw OR (life stories):ti,ab,kw OR (emic):ti,ab,kw OR (etic):ti,ab,kw OR (hermeneutics):ti,ab,kw OR (heuristic*):ti,ab,kw OR (semiotic):ti,ab,kw OR (data saturation):ti,ab,kw OR (participant observation):ti,ab,kw OR (action research):ti,ab,kw OR (cooperative inquiry):ti,ab,kw OR (co-operative inquiry):ti,ab,kw OR (field study):ti,ab,kw OR (field studies):ti,ab,kw OR (field research):ti,ab,kw OR (theoretical sample):ti,ab,kw OR (theoretical samples):ti,ab,kw OR (theoretical sampling):ti,ab,kw OR (purposive sampling):ti,ab,kw OR (purposive sample*):ti,ab,kw OR (lived experience*):ti,ab,kw OR (content analysis):ti,ab,kw OR (content analyses):ti,ab,kw OR (discourse):ti,ab,kw OR (narrative analysis):ti,ab,kw OR (narrative analyses):ti,ab,kw OR (heidegger*):ti,ab,kw OR (colaizzi):ti,ab,kw OR (spiegelberg):ti,ab,kw OR (van manen*):ti,ab,kw OR (van kaam):ti,ab,kw OR (merleau ponty):ti,ab,kw OR (husserl*):ti,ab,kw OR (Foucault or Corbin):ti,ab,kw OR (Strauss or Glaser):ti,ab,kw |
| #8 | (China):ti,ab,kw OR (People's Republic of China):ti,ab,kw OR (Mainland China):ti,ab,kw OR (Sinkiang):ti,ab,kw OR (Inner Mongolia):ti,ab,kw OR (Manchuria):ti,ab,kw |
| #9 | #3 OR #4 |
| #10 | #5 OR #6 |
|  | #1 AND #2 AND #9 AND #10 #AND #7 AND #8 |
| **万方** | |
|  | 题名或关键词:(护士 or 护理 or 照护人员 or 照护 or 照顾) and 全部:(养老院 or 养老机构 or 长期照护 or 养老护理 or 老年护理) and 全部:(压力 or 困难 or 挑战 or 障碍 or 老龄化) and 全部:(应对 or 解决 or 措施 or 方法 or 经验 or 体验 or 态度) and 题名或关键词:(质性研究 or 定性 or 半结构 or 非正式 or 焦点小组 or 面对面 or 田野工作 or 现象学 or 民族志 or 生活经验 or 内容分析 or 叙事分析 or colaizzi) |
| **中国知网** | |
|  | (TKA='护士'+'护理'+'照护人员'+'照护'+'照顾' ) AND (FT='养老院'+'养老机构'+'长期照护'+'养老护理'+’老年护理’) AND (FT='压力'+'困难'+'挑战'+'障碍'+’老龄化’) AND (FT='应对'+'解决'+'措施'+'方法'+'经验'+'体验'+'态度') AND ( TKA='质性研究'+'定性研究'+'半结构'+'非正式'+'焦点小组'+'面对面'+'田野工作'+'现象学'+'民族志'+'生活经验'+'内容分析'+'叙事分析'+'colaizzi') |
| **维普** | |
|  | (M=护士 OR 护理 OR 照护人员 OR 照护 OR 照顾 ) AND (U=养老院 OR 养老机构 OR 长期照护 OR 养老护理 OR 老年护理) AND (U=压力 OR 困难 OR 挑战 OR 障碍 OR 老龄化) AND (U=应对 OR 解决 OR 措施 OR 方法 OR 经验 OR 体验 OR 态度) AND ( M=质性研究 OR 定性研究 OR 半结构 OR 非正式 OR 焦点小组 OR 面对面 OR 田野工作 OR 现象学 OR 民族志 OR 生活经验 OR 内容分析 OR 叙事分析 OR colaizzi) |
