# Supplementary material for: Challenges and coping experiences faced by nursing staff in long-term care facilities in China: a qualitative meta-analysis
Source: Front Public Health. 2024 Jan 8;11:1302481. doi: 10.3389/fpubh.2023.1302481 (PMC10800415; doi:10.3389/fpubh.2023.1302481)
Supplement: Supplementary file 2 [file Table_2.docx]

Joanna Briggs Institute qualitative assessment and review tool

|  | Q1 | Q2 | Q3 | Q4 | Q5 | Q6 | Q7 | Q8 | Q9 | Q10 |
| --- | --- | --- | --- | --- | --- | --- | --- | --- | --- | --- |
| (Chi et al., 2020) | Yes | Yes | Yes | Yes | Yes | No | No | Yes | Yes | Yes |
| (Zhang and Li, 2021) | U | Yes | Yes | Yes | Yes | No | No | Yes | Yes | Yes |
| (Wang et al., 2017) | Yes | Yes | Yes | Yes | Yes | No | Yes | Yes | Yes | Yes |
| (Niu, 2015) | Yes | Yes | Yes | Yes | Yes | No | No | Yes | Yes | Yes |
| (Tian et al., 2019) | Yes | Yes | Yes | Yes | Yes | No | No | Yes | Yes | Yes |
| (Wang et al., 2018) | Yes | Yes | Yes | Yes | Yes | No | No | Yes | Yes | Yes |
| (Zhou et al., 2023) | Yes | Yes | Yes | Yes | Yes | No | No | Yes | Yes | Yes |
| (Li et al., 2020) | Yes | Yes | Yes | Yes | Yes | No | No | Yes | Yes | Yes |
| (Chao et al., 2021) | Yes | Yes | Yes | Yes | Yes | Yes | No | Yes | Yes | Yes |
| (Yan et al., 2020) | U | Yes | Yes | Yes | Yes | No | No | Yes | Yes | Yes |
| (Zhou et al., 2020) | Yes | Yes | Yes | Yes | Yes | No | Yes | Yes | Yes | Yes |
| (Zhang et al., 2021) | Yes | Yes | Yes | Yes | Yes | No | No | Yes | Yes | Yes |
| (Zhu et al., 2019) | U | Yes | Yes | Yes | Yes | No | No | Yes | Yes | Yes |
| (Jiang et al., 2023) | Yes | Yes | Yes | Yes | Yes | Yes | No | Yes | Yes | Yes |
| (Wei et al., 2015) | Yes | Yes | Yes | Yes | Yes | No | No | Yes | Yes | Yes |

U=Unclear
Q1. Is there congruity between the stated philosophical perspective and the research methodology?
Q2. Is there congruity between the research methodology and the research question or objectives?

Q3. Is there congruity between the research methodology and the methods used to collect data?
Q4. Is there congruity between the research methodology and the representation and analysis of data?

Q5. Is there congruity between the research methodology and the interpretation of results?
Q6. Is there a statement locating the researcher culturally or theoretically?
Q7. Is the influence of the researcher on the research, and vice- versa, addressed?
Q8. Are participants, and their voices, adequately represented?
Q9. Is the research ethical according to current criteria or, for recent studies, and is there evidence of ethical approval by an appropriate body?
Q10. Do the conclusions drawn in the research report flow from the analysis, or interpretation, of the data?
