# Supplementary material for: Challenges and coping experiences faced by nursing staff in long-term care facilities in China: a qualitative meta-analysis
Source: Front Public Health. 2024 Jan 8;11:1302481. doi: 10.3389/fpubh.2023.1302481 (PMC10800415; doi:10.3389/fpubh.2023.1302481)
Supplement: Supplementary file 3 [file Table_3.docx]

Confidence in Evidence for Qualitative Research Methodology Reviews

| Summary of review finding | Studies contributing to the review finding | CERQual assessment of confidence in the evidence | Explanation of CERQual assessment |
| --- | --- | --- | --- |
| Sources of Challenges |  |  |  |
| Multitasking | (Caho et al., 2021)、(Zahng et al., 2021)、(Tian et al., 2019)、(Yan et al., 2020)*、*(Q. Wang et al., 2017) | High confidence | Minor concerns about methodological limitations, coherence, relevance and adequacy of data. |
| Clinical emergencies | (Xi et al., 2020)、(Zhu et al., 2019)*、*(Q. Wang et al., 2017)、(Jiang et al., 2023)、(Tian et al., 2019) | High confidence | Minor concerns about methodological limitations, coherence, relevance and adequacy of data. |
| Workplace conflict | (Zhou Y. et al., 2023)、(Wei et al., 2015)、(Tian et al., 2019)、(Yan et al., 2020)*、*(Q. Wang et al., 2017) | High confidence | Minor concerns about methodological limitations, coherence, relevance and adequacy of data. |
| Demand exceeding resources | (Jiang et al., 2023)、(Caho et al., 2021)、(Xi et al., 2020)*、*(Tian et al., 2019)、(Wei et al., 2015)、(Chi et al., 2020)、 | High confidence | Minor concerns about methodological limitations, coherence, relevance and adequacy of data. |
| Occupational discrimination | (Chi et al., 2020)、(Wei et al., 2015)、(Chi et al., 2020)、(Jiang et al., 2023)、(Tian et al., 2019) | High confidence | Minor concerns about methodological limitations, coherence, relevance and adequacy of data. |
| Practical consequences |  |  |  |
| Damaged health | (Yan et al., 2020)、(Caho et al., 2021)、(Tian et al., 2019)、(Yan et al., 2020)*、*(Q. Wang et al., 2017) | High confidence | Minor concerns about methodological limitations, coherence, relevance and adequacy of data. |
| Imbalanced life | (Yan et al., 2020)、(Caho et al., 2021)、(Tian et al., 2019) | Moderate confidence | Minor concerns regarding methodological limitations and coherence. Moderate concerns regarding relevance and adequacy of data. |
| Occupational disappointment | (D. Wang et al., 2018)、(Jiang et al., 2023)、(Zhu et al., 2019) | Moderate confidence | Moderate concerns regarding methodological limitations and adequacy of data. Minor concerns about relevance and coherence. |
| Psychological influence |  |  |  |
| Compassion Fatigue | (Jiang et al., 2023)、(Tian et al., 2019)、(Zahng et al., 2021) | Moderate confidence | Moderate concerns regarding methodological limitations and adequacy of data. Minor concerns about coherence, and relevance. |
| repressed complex | (Niu, 2015)、(Tian et al., 2019)、(Yan et al., 2020)*、*(Q. Wang et al., 2017)、(Caho et al., 2021) | High confidence | Minor concerns about methodological limitations, coherence, relevance and adequacy of data. |
| Self-doubt | (W. Zhou et al., 2020)、(Niu, 2015) | Moderate confidence | Moderate concerns regarding methodological limitations. Minor concerns about coherence, relevance and adequacy of data. |
| Favorable Reactions |  |  |  |
| self adjusting | (W. Zhou et al., 2020)、(Zhou Y. et al., 2023)、(Chi et al., 2020)、(Tian et al., 2019)、(Yan et al., 2020)*、*(Q. Wang et al., 2017)、(Caho et al., 2021) | High confidence | Minor concerns about methodological limitations, coherence, relevance and adequacy of data. |
| Feel validation and belonging | (Zhang & Li, 2021)、(Wei et al., 2015) | Moderate confidence | Moderate concerns regarding methodological limitations. Minor concerns about coherence, relevance and adequacy of data. |
| Seeking support | (Zhang & Li, 2021)、(Tian et al., 2019)、(Zhou Y. et al., 2023) | Moderate confidence | Moderate attention to relevance and coherence. Methodological limitations and data adequacy were of minor concern. |
