# Supplementary material for: Challenges and coping experiences faced by nursing staff in long-term care facilities in China: a qualitative meta-analysis
Source: Front Public Health. 2024 Jan 8;11:1302481. doi: 10.3389/fpubh.2023.1302481 (PMC10800415; doi:10.3389/fpubh.2023.1302481)
Supplement: Supplementary file 4 [file Table_4.docx]

Summary of included studies

| Author | year | country | Aim | Design | data collection and analysis | Setting | Participants | Key findings/themes |
| --- | --- | --- | --- | --- | --- | --- | --- | --- |
| Chi lian | 2020 | Kunming, Yunnan Province | To understand the real psychological experience and willingness of nursing staff with college and secondary education engaged in elderly care service in nursing institutions | Phenomenological research | Face to face semi-structured in-depth interview, Colaizzi seven-step analysis | Nursing Home | nursing aides（n=12） | Multiple psychological experience, self-responsibility, sense of accomplishment, professional learning change |
| Yunjue Zhang | 2021 | Qingdao, Shandong Province | To explore the real views of nurses in long-term care facilities on caring for the elderly | Phenomenological research | Semi-structured interview, Colaizzi 7-step analysis | Medical-nursing integrated pension institutions | clinical nurses（n=13） | High working pressure; High social support; Work is meaningful; Attach importance to career development |
| Qingqing Wang | 2017 | Tianjin | To understand the working status and psychological experience of graduates majoring in geriatric nursing after they choose to work in nursing home | Phenomenological research | Face to face semi-structured interview, Colaizzi 7-step analysis | Nursing Home | clinical nurses（n=9） | Sources of pressure in nursing home work; The matching degree between the job position and the major is low; Interpersonal relationships are complex; There are few opportunities for further study. |
| Da Niu | 2015 | Wuhan, Hubei province | To explore the working experience of junior nurses in geriatric wards | Phenomenological research | Unstructured interview, Colaizzi 7-step analysis | Hospital geriatric department | clinical nurses（n=11） | Poor adaptation of clinical nursing role change, poor adaptation of working environment, lack of humanistic care ability, lack of nursing and disease observation experience |
| Ye Tian | 2019 | Nanning ,Guangxi | To explore the working and living experience of elderly caregivers | Phenomenological research | Face-to-face interview and observation, Colaizzi 7-step analysis | Nursing Home | nursing aides（n=10） | Nursing workers for the aged work long hours, excessive workload, poor working environment, narrow career development space, psychological pressure, low professional recognition. |
| Dan Wang | 2018 | Chongqing | To understand the working experience of nurses in the nursing care department of community health service center | Phenomenological research | Semi-structured in-depth interview, Colaizzi 7-step analysis | community healthcare center | clinical nurses（n=12） | Lack of correct cognition and good recognition of old-age care occupation at the beginning of employment; In the process of elderly care service encountered pressure and challenges; Lack of systematic training in professional knowledge and skills for the elderly; They are not satisfied with the salary and welfare treatment of the elderly care profession; In the process of work, the sense of identity of professional value of elderly care has gradually become prominent |
| Yiping Zhou | 2023 | Baoding City, Heibei Province | To explore the experience of dementia care among senior caregivers for the aged in China | Phenomenological research | Face-to-face semi-structured interview, Colaizzi 7-step analysis | Nursing Home | nursing aides（n=7） | Stress and challenge (poor communication, heavy burden, risk of injury), adjustment and adaptation (attitude change, sharing life, value discovery, smart communication strategies), support and expectations (emotional support, educational support, social expectations). |
| Man Li | 2018 | Jinan, Shandong Province | To explore the working experience and feelings of nurses in aged care institutions | Descriptive phenomenological research | Semi-structured interview, Colaizzi 7-step analysis | Nursing Home | clinical nurses（n=9） | The task is heavy; Great psychological pressure; Training needs are strong; The emotional experience of work |
| Zhaoming Chao | 2021 | Qinhuangdao City and Tangshan City in Hebei Province | To understand the work experience and health needs of elderly caregivers in nursing homes | Phenomenological research | One to one semi-structured interview, Colaizzi 7-step analysis | Nursing homes, welfare homes | nursing aides（n=17） | Workload, psychological stress, difficulties faced, health behaviours and recommendations for management |
| Yuanyuan Yan | 2020 | Nanning ,Guangxi | To study the nursing experience and work status of nursing assistants in nursing homes for the aged | Phenomenological research | Semi-structured interview, Colaizzi 7-step analysis | Nursing Home | nursing aides（n=15） | The elderly have low cooperation, heavy work load and weak family support |
| Wei Zhou | 2020 | Nanning ,Guangxi | To explore the deep feelings of nurses in nursing homes caring for patients with Alzheimer's disease | Phenomenological research | Face-to-face semi-structured in-depth interview, Colaizzi 7-step analysis | Nursing Home | clinical nurses（n=11） | Heavy work tasks, heavy safety responsibilities, low social recognition, complex emotional experience, few channels for emotional catharsis and confused career development |
| Ya Zhang | 2021 | Wuxi, Jiangsu, and Changsha, Hunan | To study the real experience of caregivers for senile dementia patients | Phenomenological research | Semi-structured in-depth interview, Colaizzi 7-step analysis | Nursing Home | nursing aides（n=12） | Heavy care tasks and heavy physical and psychological burdens; Emotional communication difficulties, desire to get the understanding of the elderly and family members; Lack of care skills and inability to cope with care problems; Feel dependent in care and get positive experience of care. |
| Mengqin Zhou | 2019 | Zhejiang Province | To understand the real experience of elderly caregivers at work | Phenomenological research | Semi-structured interview, unclear | Nursing Home | nursing aides（n=12） | Positive caregiving experiences, including achievement and satisfaction, intimacy and interaction, growth and identity. Negative caregiving experiences. Including complaint and worry, grievance and loss, guilt and sadness. The possible influencing factors of different caregiving experience include role identity, emotion management and professional motivation. |
| Jiang et al | 2023 | Chengdu, Sichuan Province | To understand the work status of nurses in long-term care institutions in China for elderly care | qualitative descriptive study design | Interviewing and participatory observation, content analysis | Nursing homes | clinical nurses（n=31） | nurses in long-term care institutions had insufficient man-power. nurses were moderately paid, and their salary satisfaction was low. the social identity of nurses in long-term care institutions was low |
| Wei et al | 2015 | Fuzhou, Fujian Province | To examine how nursing aides in nursing homes perceived their caring work | Exploratory qualitative description study | focus-group interview, phenomenological hermeneutic | Nursing home | nursing aides (n=24) | Two themes (positive and negative working experiences) and six sub-themes were drawn: companionship, happiness, trust, achievement, hard work, and grievance |
